# Intracranial Tuberculomas Associated with Tuberculous Meningitis

**DOI:** 10.1590/0037-8682-0440-2024

**Published:** 2025-06-02

**Authors:** Nurten Nur Aydın, Murat Aydın, Deniz Öztürk Koçakgöl, Ömer Karaşahin

**Affiliations:** 1 Erzurum Regional Training and Research Hospital, Infectious Diseases and Clinical Microbiology Department, Erzurum, Turkey.; 2 Erzurum Regional Training and Research Hospital, Department of Radiology, Erzurum, Turkey.

**FIGURE 1:**
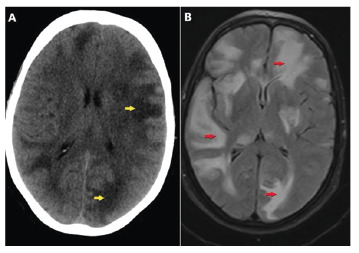
Hypodense edema in the white matter on the non-contrast axial brain tomography image **(A)**. Hyperintense edema in the white matter of the brain on the axial T2-weighted inversion recovery scan (FLAIR) image **(B)**.

**FIGURE 2:**
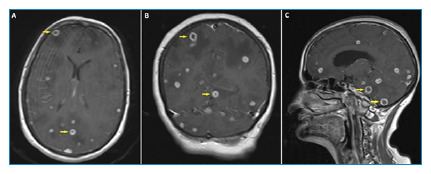
The axial **(A)**, coronal **(B)**, and sagittal **(C)** images of contrast-enhanced T1-weighted magnetic resonance imaging showing the foci of abscesses (tuberculomas) with peripheral enhancement.

A 58-year-old woman presented to the emergency department with fever, impaired consciousness, and seizures. Quadruple antituberculosis therapy for pulmonary tuberculosis was commenced and continued for one month. Physical examination revealed a fever of 37.7°C and stiffness of the neck. The C-reactive protein (CRP) levels (17.5 mg/L) and erythrocyte sedimentation rate (48 mm/h) were elevated; however, the remaining parameters were within normal limits. Previous sputum cultures confirmed the presence of *Mycobacterium tuberculosis*. Noncontrast computed tomography (CT) of the brain and magnetic resonance imaging (MRI) revealed white matter edema (Figure 1). Contrast-enhanced MRI revealed peripheral enhancement of the abscess foci (tuberculomas) (Figure 2).

The patient was diagnosed with tuberculous meningitis and related intracranial tuberculoma. Quadruple anti-TB therapy was commenced in addition to the administration of dexamethasone and mannitol to control intracranial pressure. Fluid and electrolyte management was also commenced. The lesions exhibited no significant regression following 6 months of therapy. Surgery was performed, and pathological examination confirmed the presence of necrotizing granulomatous inflammation associated with tuberculosis.

Tuberculomas are granulomatous masses arising from the hematogenous spread of *M. tuberculosis* ^1^ . These are hallmark features of severe extrapulmonary tuberculosis, present in approximately 5% of all cases of tuberculosis^2^. Imaging plays a crucial role in the early detection and differentiation of tuberculomas from other intracranial pathologies, thereby facilitating timely intervention and preventing long-term complications^3^.
